# Challenges and Limitations in Molecular Testing of Resected Non-Small Cell Lung Cancer Specimens

**DOI:** 10.3390/cimb48040419

**Published:** 2026-04-18

**Authors:** Nikolaos Korodimos, Ioannis Tomos, Periklis Foukas, Konstantinos Kontzoglou, Anna Koumarianou, Ilias Santaitidis, Konstantinos Kostopanagiotou, Sofoklis Mitsos, Anastasios Moisiadis, Periklis Tomos

**Affiliations:** 1 University Thoracic Surgery Clinic, Attikon University General Hospital, 124 62 Athens, Greece; korodimos@yahoo.com (N.K.); uni.drhgs@gmail.com (I.S.); kostop@hotmail.co.uk (K.K.); sophocmit@yahoo.gr (S.M.); periklistomos@hotmail.com (P.T.); 2 Pulmonary Department, “Sotiria” Athens Hospital for Thoracic Diseases, 115 27 Athens, Greece; 3 University Laboratory of Histopathology, Attikon University General Hospital, 124 62 Athens, Greece; pfoukas@yahoo.com; 4 University Surgical Clinic, Laiko General Hospital of Athens, 115 27 Athens, Greece; kckont@med.uoa.gr; 5 University Fourth Department of Internal Medicine, Attikon University General Hospital, 124 62 Athens, Greece; akoumari@yahoo.com

**Keywords:** non-small cell lung cancer (NSCLC), molecular testing, next-generation sequencing (NGS), immunohistochemistry (IHC), fluorescence in situ hybridization (FISH), liquid biopsy, formalin-fixed paraffin-embedded (FFPE), targeted therapy, multi-omics, precision oncology

## Abstract

Non-small cell lung cancer (NSCLC) accounts for nearly 85% of lung cancer cases and remains a leading cause of cancer-related mortality worldwide. Advances in molecular diagnostics and targeted therapies have transformed treatment paradigms, yet the integration of molecular testing into routine care for resected NSCLC specimens continues to face significant challenges. This review outlines the technical, clinical, and systemic barriers that limit the effectiveness of molecular testing. Key considerations include tissue quality, the limitations of formalin-fixed paraffin-embedded (FFPE) samples, and the comparative roles of conventional methods—such as immunohistochemistry (IHC), fluorescence in situ hybridization (FISH), and reverse transcription polymerase chain reaction (RT-PCR)—versus next-generation sequencing (NGS). We also discuss the prevalence and clinical relevance of common genomic alterations, including TP53, KRAS, EGFR, and ALK, as well as their impact on prognosis and treatment selection. Real-world obstacles such as accessibility, reimbursement, delays in testing, interdisciplinary coordination, and sample adequacy are critically examined. Emerging innovations—including multi-omics integration, spatial profiling, liquid biopsy, artificial intelligence, and novel targeted therapies—offer opportunities to overcome current limitations and improve patient outcomes. Finally, practical recommendations are proposed to optimize tissue handling, testing algorithms, and access to precision-guided therapies. By addressing these challenges, molecular testing in NSCLC can be more effectively leveraged to personalize treatment strategies and enhance survival outcomes.

## 1. Introduction

NSCLC refers to about 85% of all lung cancer instances, still remains a leading cause of cancer-related mortality globally, with over 2 million deaths annually [1,2]. Notwithstanding advances in early detection, surgery, radiotherapy, and systemic treatments, the five-year survival rate for NSCLC remains below 20% in most countries [3]. During the past 20 years, considerable progress in molecular pathology and targeted therapies has transformed management, permitting accuracy in oncology approaches assisting in the improvement of patient outcomes [3,4].

Molecular profiling has become a cornerstone in the diagnosis and treatment planning of NSCLC. Presently updated international guidelines recommend testing for actionable alterations, including EGFR, ALK, ROS1, BRAF, KRAS, RET, MET, ERBB2/HER2, and NTRK [4,5]. Identification of these alterations allows the use of targeted remedies, that have been proven to meaningfully prolong progression-free and overall survival in accordance with conventional chemotherapy [4,6]. In accordance, real-world studies disclose that molecular testing is often underutilized, delayed, or restricted to a subset of biomarkers, consequentially resulting in missed opportunities for optimized, individualized treatment [6,7].

Factually and through the years of evolution of analytical methods, molecular testing relied on single-gene methods, such as immunohistochemistry (IHC), fluorescence in situ hybridization (FISH), and reverse transcription polymerase chain reaction (RT-PCR) [4]. While these methods are reliable, they are limited by tissue requirements, throughput, and the scope of detectable alterations. NGS has made it possible to analyse many genes at once with just a little amount of tissue, which makes it more likely that changes that can be acted on will be found [3,4]. However, NGS is not generally available, and its combination with traditional methods is often necessary to optimise detection rates [4,6].

In addition to technical issues, molecular testing faces problems with sample quality, limits of formalin-fixed paraffin-embedded (FFPE) tissue, and the need for surgical, pathology, and oncology teams to work together [4,7]. Additionally, variations in healthcare systems, payment regulations, and laboratory infrastructure exacerbate the challenges of prompt and thorough molecular analysis [3,6].

Due to these obstacles, it is crucial to comprehend the prevalence, constraints, and practical utility of genetic testing to inform the treatment of resected NSCLC tissues. This research seeks to investigate the technological hurdles, detection rates, and prospective enhancements in molecular testing procedures for NSCLC, aiming to provide pragmatic insights for the augmentation of patient care and accessibility to targeted medicines [3,6].

Although general principles of molecular testing apply across different NSCLC specimen types, the present review specifically focuses on surgically resected non-small cell lung cancer specimens. With the increasing incorporation of molecular profiling into early-stage and resectable disease, the clinical relevance of biomarker testing has expanded beyond the metastatic setting. In resected specimens, the main challenges differ from those encountered in small biopsies and are more closely related to tissue quality, pre-analytical handling, tumor heterogeneity, tumor cellularity, and workflow efficiency. References to biopsy-based or advanced-stage NSCLC literature are included where relevant to provide contextual or comparative insight; however, the primary emphasis of this review remains on the distinct diagnostic and practical considerations associated with resected material.

Given the broad scope of this review and the heterogeneity of the literature considered, the aim is not to provide a systematic or quantitative synthesis of outcomes, but rather to present a clinically oriented overview of the technical, biological, and implementation-related challenges associated with molecular testing in resected NSCLC.

## 2. Technical Aspects of Molecular Testing

Referring to non-small cell lung cancer (NSCLC), therapeutic approaches and molecular testing are equivalently essential for the creation of personalized strategies and prognoses. Considering biological material type and the quality and testing methodology can greatly affect the accuracy and reliability of these results [8].

### 2.1. Sample Types and Quality Considerations

The type and quality of samples are crucial for conducting effective molecular testing and producing fruitful results. Matters of cellularity, the way samples are preserved, and the accessibility of tissues may considerably influence the functionality and reliability of subsequent tests [6]. Surgically resected tissues remain the gold standard for molecular analysis of non-small cell lung cancer (NSCLC) because they generally contain larger and more representative tumour regions. In the resected NSCLC setting, these pre-analytical and processing factors acquire particular importance, as specimen size and tissue volume introduce logistical constraints that are distinct from those encountered in small biopsy samples. Larger resections may be associated with prolonged and variable cold ischemia times and heterogeneous formalin penetration, resulting in uneven nucleic acid preservation across different tumor regions. In addition, resection specimens frequently exhibit spatially variable tumor cellularity and pronounced intratumoral heterogeneity, making pathologist-guided selection of representative, tumor-rich blocks critical to avoid sampling bias and false-negative results. Finally, post-neoadjuvant resections may contain extensive necrosis or fibrosis, further limiting viable tumor for extraction and increasing the risk of inadequate material for NGS. Such samples often preserve tumour heterogeneity more effectively than biopsies, allowing for a fuller depiction of clonal and subclonal alterations. This increases diagnostic confidence and offers clinicians the opportunity to retest or extend the molecular panel if new therapeutic questions arise. Ensuring proper handling is fundamental; even short delays in fixation or inconsistent processing across different areas of a large resected specimen may lead to uneven preservation, ultimately lowering nucleic acid quality and affecting test accuracy. This is particularly relevant in molecular assays such as IHC, FISH, NGS, which depend on the structural and chemical integrity of the sample. Such factors may significantly affect nucleic acid quality and the reliability of downstream molecular testing. In addition, intra-tumor heterogeneity represents a significant challenge in resected tumors, particularly in lesions exceeding 3 cm in size. Mixed histological patterns and spatially distinct tumor subclones may coexist within the same specimen, raising the possibility that molecular testing performed on a single tumor block may fail to capture clinically relevant subclonal alterations present in other regions of the tumor [9]. Surgically resected NSCLC specimens encompass a spectrum of procedures, including wedge resections, segmentectomies, lobectomies, pneumonectomies, and sleeve resections. While these specimen types differ in anatomical extent and surgical complexity, the primary challenges for molecular testing are largely driven by specimen size, tissue volume, and processing logistics rather than by the specific surgical procedure itself. Larger and more complex resections are associated with increased cold ischemia times, heterogeneous fixation, and greater intratumoral heterogeneity, whereas smaller resections may be limited by reduced tumor cellularity and sampling constraints. These factors must be considered during specimen handling to ensure representative and reliable molecular analysis. Pre-analytical handling remains a critical determinant of molecular testing success in resected NSCLC specimens. Variability in cold ischemia time and formalin fixation duration may significantly affect nucleic acid integrity, particularly in large surgical samples with heterogeneous fixation patterns. In cases of chest wall invasion requiring decalcification, the use of strong acidic decalcifying agents can severely compromise DNA and RNA quality, rendering specimens unsuitable for molecular analysis. Furthermore, in patients treated with neoadjuvant therapy, surgical specimens may predominantly consist of necrosis or fibrosis, posing challenges for pathologic response assessment and often limiting the availability of sufficient viable tumor cells (commonly ≥ 20% tumor cellularity) required for reliable NGS-based testing [7,10]. Estimating tumour content is equally essential, as it guides macrodissection strategies and helps ensure that the extracted nucleic acids meet the minimal input and purity thresholds required for sensitive mutation detection [11]

FFPE tissue is among the most commonly used preparations in clinical practice because it enables long-term storage while retaining morphological detail suitable for microscopic review. Despite its advantages, the FFPE process introduces several limitations that can significantly affect molecular assays. Formalin crosslinking, nucleic acid fragmentation, and the introduction of artefactual sequence changes may reduce the fidelity of downstream analyses, particularly when working with low-frequency variants or fragile RNA targets. These biochemical alterations can hinder polymerase efficiency, compromise hybridization-based assays, and reduce the accuracy of NGS, especially in genomic regions rich in cytosine residues where deamination artefacts frequently occur. Sampling challenges may further exacerbate these issues: diminutive biopsy specimens or FFPE blocks with heterogeneous tumour distribution may yield insufficient tumour-rich areas for extraction, heightening the risk of false negatives. To mitigate these risks, laboratories increasingly adopt standardized fixation protocols, automated tissue processors, and robust pre-analytical quality control systems designed to quantify nucleic acid integrity before sequencing. Parameters such as DNA DIN, RNA RIN, and amplifiability indices provide early indications of sample suitability and help avoid wasted sequencing runs. Optimised extraction techniques, combined with careful histopathological evaluation of tumour-rich areas, can substantially improve the reliability of molecular results and support more confident clinical decision-making [8].

### 2.2. Conventional Techniques

Standard molecular techniques continue to be indispensable in diagnosing non-small cell lung cancer (NSCLC) [5]. Their accessibility, quick processing times, and minimal tissue requirements make them first-line diagnostic tools. For instance, IHC techniques examine clinically significant ALK, ROS1, and PD-L1 biomarkers to determine cancer tissue protein expression levels [12]. Visualized and captured image markers identify target proteins in tissues using antibodies; the method then uses chromogenic or fluorescent detection methods. Its speed and low cost make it ideal for small biopsies [12]. Unfortunately, IHC method antibodies, epitope preservation, and overall assay standardization heavily contribute to limitations in sensitivity and specificity in results [12]. In positive IHC results, other methods, such as FISH methods, are frequently required [12].

FISH is the gold standard for identifying rearrangements, amplifications, and other copy number alterations in NSCLC [11]. Its specificity allows for the evaluation of chromosomal alterations such as ALK, ROS1, and RET rearrangements [11]. FISH is the only technique that offers single-cell resolution. FISH is very specific, but the technique is very demanding and laborious. It requires high quality tissues and cannot be used for detection of point mutations or small insertions [11]. FISH also cannot be used for small tissues.

RT-PCR enables the sensitive detection of gene fusions and specific mutations at the RNA level, such as NTRK fusions [10]. RT-PCR offers high sensitivity and rapid results but is typically restricted to predefined targets, making it less suitable for broad profiling or discovery of novel alterations [10].

### 2.3. Next-Generation Sequencing

Next-generation sequencing has assisted in the revolution of molecular diagnostics in NSCLC by allowing the instantaneous analysis of numerous genes or genomic regions from tumor DNA or RNA [13]. This methodology, with its efficient and high-throughput capabilities, facilitates the detection of point mutations, insertions and deletions, copy number variations, and gene fusions in a single assay [13]. NGS workflows generally involve sample preparation, construction of data libraries, sequencing, and bioinformatic analysis. Platforms vary widely, ranging from targeted panels focused on clinically actionable genes to whole-exome or whole-genome sequencing, each differing in throughput capacity, read length, and coverage depth [13]. Targeted panels are typically used in routine clinical practice because they offer high sensitivity for low-frequency variants and cover the most therapeutically relevant alterations. In contrast, whole-exome or whole-genome sequencing provides broader coverage and more extensive molecular insights but is less practical for most diagnostic laboratories due to cost, turnaround time, and data interpretation challenges.

Next-generation sequencing offers several advantages compared with conventional methodologies. Its multiplexing capability enables the simultaneous detection of multiple actionable alterations from limited tissue, which is particularly important in NSCLC, where driver mutations such as EGFR, KRAS, ALK, and BRAF may coexist or emerge sequentially during disease progression [13,14]. Moreover, NGS provides quantitative information about variant allele frequencies and can detect co-occurring mutations that traditional methods may miss, offering a more complete understanding of tumour biology and heterogeneity [14]. These insights are increasingly relevant for treatment resistance mechanisms, especially in the era of sequential targeted and immunotherapies.

However, NGS is not without limitations [9,13]. Longer turnaround times compared with single-gene assays may delay therapeutic decision-making, particularly when testing is outsourced to external laboratories. Costs remain substantial for many institutions, limiting access and creating disparities in testing availability between academic and community settings. NGS platforms also depend heavily on specialized personnel and robust bioinformatics pipelines capable of handling large datasets and distinguishing true variants from sequencing artefacts [9]. Sensitivity is influenced by tumour cellularity, DNA or RNA integrity, and sequencing depth, meaning that suboptimal samples may yield incomplete or inconclusive results. Furthermore, the interpretation of variants of uncertain significance (VUS) poses ongoing clinical challenges and may complicate therapeutic planning [9]. Although the present review focuses primarily on diagnostic workflows, pre-analytical and analytical limitations, and methodological challenges rather than exhaustive molecular profiling, the clinical impact of major driver alterations is consistently acknowledged throughout the manuscript. Representative oncogenic drivers such as EGFR, ALK, and KRAS are discussed in the context of testing strategies, assay selection, interpretation challenges, and their implications for therapeutic decision-making in resected NSCLC. In particular, the review emphasizes how the biological characteristics of these alterations, including mutation type, allelic frequency, and co-occurring genomic events, intersect with specimen-related factors such as tumor cellularity, heterogeneity, and tissue quality. This integrated approach highlights the clinical relevance of molecular alterations while maintaining a diagnostic- and workflow-oriented perspective. Continued refinement of bioinformatic algorithms, standardization of reporting frameworks, and improved access to reference databases are essential to fully overcome these issues and ensure reliable NGS implementation in routine clinical workflows.

### 2.4. Integration of Conventional and NGS Approaches

Optimal molecular testing strategies often involve an integrative approach that combines conventional techniques with NGS [11]. This strategy balances the need for rapid results, cost-effectiveness, and comprehensive profiling. For example, IHC can serve as a rapid screening tool for protein expression, guiding reflex testing with FISH or NGS for confirmation and broader molecular analysis [11]. Such an approach maximizes the utility of limited tumour material while enabling timely therapeutic decisions. Integrating conventional and NGS methods ensures that patients receive accurate and comprehensive molecular characterization, which is essential for personalized treatment planning and optimizing clinical outcomes in NSCLC [11].

## 3. Prevalence and Clinical Relevance of Molecular Alterations

Non-small cell lung cancer (NSCLC) exhibits significant molecular heterogeneity [14], influencing prognosis and guiding treatment strategies. Recent advancements in molecular diagnostics have facilitated the identification of various genetic alterations that serve as predictive and prognostic biomarkers, enabling personalized therapeutic approaches [14].

### 3.1. Common Mutations in NSCLC

Non-small cell lung cancer represents a heterogeneous group of tumors comprising distinct histological subtypes, most commonly adenocarcinoma and squamous cell carcinoma, which frequently exhibit different molecular profiles. Adenocarcinomas are more often associated with actionable driver alterations such as EGFR mutations, ALK or ROS1 rearrangements, and KRAS mutations, whereas squamous cell carcinomas typically display a lower prevalence of these drivers and are more commonly characterized by alterations in TP53, CDKN2A, and other tumor suppressor genes. In resected specimens, mixed histological patterns may coexist within the same tumor, further complicating molecular interpretation and underscoring the importance of correlating histopathological findings with comprehensive genomic profiling [5,11].

#### 3.1.1. TP53

TP53 is among the most frequently altered genes reported in NSCLC cohorts, with substantial prevalence across multiple histologic and molecular subgroups [15]. These alterations have been associated with poorer prognosis and may also influence treatment responses, including those to immune checkpoint inhibitors, although the magnitude of these associations may vary across study populations and clinical settings [16].

#### 3.1.2. KRAS and KRAS G12C

KRAS mutations are commonly reported in NSCLC, although their frequency varies across histologic subtypes and study populations; within this group, the G12C variant represents a clinically important molecular subset [4]. The development of KRAS G12C inhibitors has provided new therapeutic options for patients harboring this mutation. The development of KRAS G12C inhibitors has provided new therapeutic options for patients harboring this mutation. Importantly, currently approved targeted therapies are limited to the KRAS G12C subtype and are not applicable to other KRAS variants [17,18].

#### 3.1.3. EGFR

EGFR mutation frequency varies substantially according to ethnicity, geographic region, and histologic subtype, with higher rates reported in Asian and adenocarcinoma-enriched cohorts. The most common sensitizing alterations include exon 19 deletions and exon 21 L858R mutations [19]. These alterations predict sensitivity to EGFR tyrosine kinase inhibitors and have important implications for treatment selection across advanced and, increasingly, resected early-stage disease [3].

#### 3.1.4. ALK, ROS1, BRAF, MET, RET, HER2, NTRK

ALK and ROS1 rearrangements, as well as alterations involving BRAF, MET, RET, HER2, and NTRK, represent less frequent but clinically actionable molecular subsets of NSCLC [3,10]. Targeted therapies directed against these alterations have shown clinical activity in selected molecular populations, although the strength of evidence and degree of implementation vary across biomarkers and treatment settings [10]. Although this review places particular emphasis on TP53, KRAS, EGFR, and ALK because of their biological and clinical relevance, these alterations do not fully represent the broader spectrum of actionable biomarkers currently involved in NSCLC management. Additional clinically relevant alterations, including ROS1 rearrangements, BRAF V600E mutations, MET exon 14 skipping, RET fusions, HER2 mutations, and NTRK fusions, also contribute to treatment stratification and may be particularly important in selected molecular and histologic subsets. A detailed discussion of all actionable biomarkers, however, is beyond the scope of the present review, which is intended to provide a broader overview of the technical, clinical, and implementation-related challenges of molecular testing in resected NSCLC.

### 3.2. Targetable vs. Non-Targetable Alterations

Molecular alterations in NSCLC are categorized as targetable or non-targetable based on the availability of effective therapies. Targetable alterations, such as EGFR, ALK, ROS1, BRAF V600E, MET exon 14, RET, HER2, KRAS G12C, and NTRK fusions, enable the use of genotype-directed therapies and have been associated with improved outcomes in selected molecular subgroups of NSCLC [3]. Importantly, many of the actionable genomic alterations identified through NGS—including EGFR mutations, ALK and ROS1 rearrangements, BRAF V600E mutations, MET exon 14 skipping, RET fusions, and KRAS G12C—are primarily targeted by small-molecule inhibitors, most commonly tyrosine kinase inhibitors, which form the backbone of precision-guided systemic therapy in NSCLC [5]. Non-targetable alterations, like most TP53 mutations, currently lack specific therapies but provide prognostic information and may influence treatment decisions indirectly, including suitability for immunotherapy or combination regimens [20].

As according to Özgür et al. (2025) the distribution of frequently observed molecular alterations in NSCLC across all cohorts is summarized in Table 1 [4]. TP53 mutations are the most common (52.9%), followed by KRAS (20.0%), EGFR (8.6%), STK11 (8.6%), PIK3CA (7.1%), CDKN2A (7.1%), ALK (5.7%), ERBB2 (4.3%), RB1 (4.3%), and ATRX (4.3%). These results highlight the heterogeneity of NSCLC and the importance of comprehensive molecular profiling for targeted therapy selection. From a clinical perspective, comprehensive molecular profiling in resected non-small cell lung cancer extends beyond mutation detection and increasingly informs individualized patient management. The identification of targetable driver alterations and clinically relevant co-mutations may influence postoperative surveillance strategies, eligibility for adjuvant or perioperative targeted therapies, and enrollment in molecularly driven clinical trials. Moreover, understanding non-targetable alterations with prognostic significance supports risk stratification and multidisciplinary decision-making. As therapeutic options continue to expand, clinically oriented interpretation of molecular results is essential to translate genomic data into meaningful improvements in patient outcomes [5,14,20].

### 3.3. Impact on Prognosis and Treatment Selection

In resected NSCLC, the presence and type of molecular alterations may inform prognostic assessment and support personalized postoperative and perioperative treatment strategies. Although much of the available evidence on the prognostic and therapeutic impact of molecular alterations derives from advanced-stage NSCLC, these findings are increasingly relevant to resected disease as biomarker testing expands into adjuvant and perioperative treatment decision-making. PD-L1 expression remains a key biomarker for immunotherapy selection in NSCLC, its predictive value is strongly influenced by co-occurring genomic alterations. In particular, mutations in STK11 and KEAP1 have been associated with an immunologically “cold” tumor microenvironment and primary resistance to immune checkpoint inhibitors, even in tumors exhibiting high PD-L1 expression. This highlights a critical limitation of relying on PD-L1 status alone for treatment decisions. Consequently, integrated molecular profiling that incorporates PD-L1 expression together with relevant genomic alterations is increasingly necessary to accurately predict immunotherapy benefit and to guide personalized treatment strategies in both advanced and resected NSCLC [6]. Current clinical practice guidelines increasingly support the integration of molecular testing into early-stage NSCLC management. Recent NCCN and ESMO recommendations now advocate EGFR mutation testing in patients with resected Stage IB–IIIA NSCLC to guide eligibility for adjuvant targeted therapies, as well as PD-L1 assessment in Stage II–IIIA disease [21]. This paradigm shift from a predominantly metastatic testing framework toward early-stage molecular profiling underscores the relevance of optimized testing strategies in the resected setting. However, implementation remains heterogeneous, as regional reimbursement policies and financial constraints may limit access to comprehensive NGS testing in early-stage disease, with coverage in some healthcare systems still primarily restricted to metastatic cases. EGFR-mutant and ALK-rearranged NSCLCs are established molecular subsets for which tyrosine kinase inhibitors have demonstrated major therapeutic benefit, although the approved use and evidence base differ by disease stage and treatment setting [3]. In contrast, targeted therapeutic options for KRAS-mutant disease have historically been limited, with current approved KRAS-directed therapies mainly restricted to the KRAS G12C subtype [17]. Additionally, the identification of co-mutations or concurrent alterations can modify prognosis and affect therapeutic efficacy, emphasizing the importance of comprehensive molecular profiling for treatment planning [22]. For example, alterations involving TP53, STK11, or KEAP1 have been associated with more aggressive disease biology and variable responses to targeted agents or immunotherapy [22]. Recognizing these molecular contexts allows clinicians to better stratify patients, anticipate resistance patterns, and tailor treatment sequencing, particularly as combination and adjuvant strategies continue to evolve. From a clinical standpoint, the interpretation of molecular alterations in NSCLC extends beyond the identification of individual driver mutations. The presence of co-occurring genomic alterations, such as TP53, STK11, or KEAP1 mutations, may significantly influence prognosis and treatment response, particularly in the context of targeted therapies and immunotherapy [23]. Emerging evidence suggests that these co-mutations are associated with more aggressive tumor biology, altered tumor microenvironment, and reduced benefit from certain systemic treatments. In resected NSCLC, molecular profiling increasingly informs postoperative management, including risk stratification, selection of adjuvant or perioperative targeted therapies, and eligibility for molecularly driven clinical trials [24]. As treatment paradigms evolve, a comprehensive and clinically oriented interpretation of molecular findings is essential to translate genomic data into personalized therapeutic strategies and improved patient outcomes [20]. In resected NSCLC, the clinical relevance of molecular testing is increasingly supported by outcome-based evidence from biomarker-selected perioperative treatment strategies. For example, adjuvant osimertinib has demonstrated a significant overall survival benefit in completely resected EGFR-mutated stage IB–IIIA NSCLC, supporting the value of EGFR testing in early-stage disease. Similarly, adjuvant alectinib has shown a marked disease-free survival benefit in resected ALK-positive stage IB–IIIA NSCLC. These findings illustrate that, in selected molecular subgroups, biomarker testing in resected disease may directly influence postoperative treatment decisions and improve clinically meaningful outcomes [25]. Similarly, perioperative immunotherapy has also shown clinically meaningful benefit in resectable NSCLC, with studies such as CheckMate 816 and KEYNOTE-671 demonstrating improvements in event-free survival and, more recently, overall survival, further underscoring the clinical importance of biomarker-directed treatment strategies in earlier-stage disease [26,27,28]. At the same time, the present review does not aim to provide pooled quantitative estimates of survival benefit or treatment effect specifically in resected NSCLC; rather, this section is intended as a qualitative overview of the available evidence linking molecular findings to prognostic assessment and treatment selection

## 4. Real-World Challenges in Molecular Testing

Despite major advances in molecular diagnostics, the application of testing in resected NSCLC specimens remains limited by real-world barriers [6]. These challenges reduce testing rates, delay access to targeted therapies, and ultimately affect patient outcomes. The magnitude and clinical impact of these barriers may vary substantially across healthcare systems, institutional settings, reimbursement environments, and laboratory infrastructure.

### 4.1. Accessibility and Reimbursement Issues

Access to comprehensive molecular testing varies considerably between regions and healthcare systems. In high-income countries, NGS and multiplexed assays are increasingly incorporated into clinical practice, but in many parts of the world, testing is limited to a narrow panel of biomarkers due to infrastructure, cost, and reimbursement constraints [5]. Even in developed settings, reimbursement policies may not cover all actionable biomarkers, leading to selective testing and missed therapeutic opportunities [6]. These disparities underscore the need for equitable healthcare policies and the development of cost-effective testing algorithms that ensure broad patient access.

### 4.2. Timing of Molecular Testing

Timely molecular testing is essential for guiding treatment selection, especially as targeted therapies are most effective when initiated early in the treatment course. However, delays are common due to logistical issues such as sample transfer, batching of NGS runs, and extended turnaround times for sequencing analysis [7]. In some cases, results are not available before clinical decision-making, leading to the initiation of empirical chemotherapy rather than precision-guided therapy [6]. Streamlined workflows and reflex testing strategies, where molecular testing is initiated automatically upon diagnosis, may help to reduce delays and improve treatment allocation.

### 4.3. Interdisciplinary Coordination

Effective molecular testing requires close collaboration between surgeons, pathologists, oncologists, and molecular laboratories. Breakdowns in communication or unclear responsibility for ordering tests often contribute to underutilization [11]. Multidisciplinary tumor boards and molecular pathology review committees can facilitate interpretation of results and ensure that findings are translated into clinical practice [14]. Establishing standardized reporting systems and structured communication pathways remains critical for integrating molecular data into real-world oncology practice.

### 4.4. Sample Adequacy and Repeat Biopsies

Adequate tissue sampling is a frequent barrier in NSCLC molecular testing. Although resected specimens usually provide sufficient material, issues such as necrosis, poor fixation, or low tumor cellularity may compromise nucleic acid quality [8]. In some cases, insufficient tissue necessitates repeat biopsies, which increase patient risk and healthcare costs [7]. Furthermore, overuse of tissue for histopathological and immunohistochemical studies may limit the amount available for molecular testing. This challenge becomes more pronounced as the number of required biomarkers continues to expand, placing additional pressure on laboratories to balance diagnostic and molecular needs. Adoption of optimized tissue-handling protocols, macrodissection for tumor enrichment, and incorporation of minimally invasive alternatives such as liquid biopsy may mitigate these limitations [3]. Liquid biopsy, in particular, offers a valuable supplementary source of tumor-derived nucleic acids when tissue samples are inadequate and may reduce the reliance on repeat invasive procedures while still enabling clinically meaningful genomic profiling. Its broader applications in longitudinal monitoring and complementary molecular assessment are discussed below. In addition, the increasing adoption of comprehensive genomic panels has intensified the need for high-quality input material, as broader assays typically require larger quantities of intact DNA or RNA. Laboratories must therefore implement stringent pre-analytical review processes, including systematic tumour-area marking by pathologists and standardized criteria for evaluating sample adequacy prior to extraction. The use of rapid on-site evaluation (ROSE) during biopsy procedures has also been proposed as a strategy to ensure that sufficient cellular material is collected from the outset, thereby reducing the likelihood of non-diagnostic samples. As molecular testing becomes more deeply embedded in routine care, collaborative communication between surgeons, pulmonologists, and pathologists will be essential to optimize sampling practices and secure the material necessary for accurate genomic profiling.

## 5. Advances and Future Perspectives

Ongoing innovations in molecular diagnostics and therapeutics are increasingly reshaping the management of resected and resectable NSCLC [14]. Much of the evidence supporting these emerging approaches derives from advanced-stage NSCLC; however, their potential relevance to resected disease is increasing as molecular testing expands into earlier-stage and perioperative care. Beyond conventional genotyping, multi-omics integration, minimally invasive diagnostics, and artificial intelligence (AI) are emerging as potentially transformative tools. In the resected setting, these approaches may improve specimen characterization, support risk stratification, and refine postoperative or perioperative treatment selection. Because these areas are evolving rapidly, their clinical role and implementation in resected NSCLC are likely to continue changing as additional evidence emerges.

### 5.1. Multi-Omics and Spatial Profiling Approaches

Multi-omics approaches combine genomic, transcriptomic, epigenomic, and proteomic data to provide a more comprehensive understanding of NSCLC biology. This integrated profiling allows clinicians to identify co-occurring alterations, such as TP53 mutations, which carry prognostic implications [3]. Spatial profiling, which preserves the tumor microenvironment context, is further refining biomarker discovery by highlighting intratumoral heterogeneity and immune interactions [5]. These technologies are expected to support more precise stratification of patients for targeted or combination therapies. In addition, multi-omic datasets can reveal pathway-level disruptions or molecular patterns that may not be apparent through single-modality testing alone, thereby uncovering new therapeutic targets. As analytical platforms continue to mature and become more accessible, the integration of multi-omics into routine clinical workflows is likely to enhance personalized treatment planning and contribute to more accurate prediction of treatment response and disease progression.

### 5.2. Liquid Biopsy and Minimally Invasive Techniques

Liquid biopsy has emerged as a valuable complement to tissue-based testing, particularly when surgical or biopsy samples are limited. Circulating tumor DNA (ctDNA) analysis enables dynamic monitoring of tumor evolution, early detection of resistance mutations, and assessment of minimal residual disease [3]. Compared with traditional re-biopsies, liquid biopsy is less invasive and more suitable for repeated sampling, making it well-positioned to support adaptive treatment strategies in clinical practice [6].

### 5.3. Artificial Intelligence in Molecular Testing

AI-based tools are increasingly being explored across lung cancer radiology, digital pathology, and multi-modal data interpretation, with potential applications in biomarker prediction, image analysis, and workflow optimization. These technologies may also help reduce observer variability and improve the integration of complex molecular and imaging datasets [29,30]. AI-driven predictive models can also help identify patients most likely to benefit from targeted therapies or immunotherapy, potentially reducing time to treatment and optimizing outcomes [6]. Beyond prediction, AI tools are increasingly used to detect subtle radiologic or histologic features associated with aggressive behaviour, resistance mechanisms, or specific genomic alterations. Such insights may support earlier clinical decision-making and assist clinicians in prioritizing patients for further molecular testing or clinical trial enrollment, thereby enhancing the overall efficiency of precision oncology workflows.

### 5.4. Novel Targeted Therapies and Combination Regimens

The therapeutic landscape of NSCLC has expanded with new agents targeting uncommon driver alterations. For example, larotrectinib has demonstrated remarkable efficacy in NTRK fusion-positive cancers, establishing a paradigm of tumor-agnostic therapy [10]. Similarly, the development of KRAS(G12C) inhibitors such as AMG 510 has opened new avenues for targeting historically “undruggable” mutations [17,31,32]. Combination regimens incorporating targeted therapy with immunotherapy or chemotherapy are being actively investigated to overcome limited durability of single-agent responses and broaden therapeutic benefit [14].

### 5.5. Overcoming Drug Resistance

Drug resistance remains a major obstacle to durable responses in NSCLC. Mechanisms such as secondary mutations, bypass pathway activation, and phenotypic transformation drive treatment failure [33]. Advances in next-generation inhibitors designed to overcome resistance mutations, as well as adaptive treatment strategies guided by ctDNA monitoring, are under development [3]. In addition, rational combinations targeting multiple pathways are being explored to preempt or delay resistance. These strategies highlight the importance of longitudinal molecular monitoring as an integral part of NSCLC management. As our understanding of tumor evolution improves, it is becoming evident that resistance is rarely driven by a single event; instead, it reflects a dynamic interplay of genomic instability, selective therapeutic pressure, and microenvironmental influences. Incorporating serial molecular assessments into routine care may therefore allow earlier detection of emerging resistance mechanisms and support more timely therapeutic adjustments.

## 6. Practical Recommendations and Best Practices

As molecular testing becomes increasingly central to the management of resected NSCLC, practical strategies are needed to overcome technical and systemic barriers [11]. For practical clarity, the main recommendations emerging from this review can be grouped into four implementation domains: pre-analytical optimization, testing workflow, multidisciplinary interpretation, and equitable access to biomarker-directed care.

### 6.1. Optimizing Tissue Collection and Processing

Proper tissue collection and processing are foundational for reliable molecular testing. Pathologists should prioritize tissue preservation during surgical resections and biopsies to avoid compromising nucleic acid quality. Standardized fixation protocols, avoidance of excessive decalcification, and judicious use of tissue for both histology and molecular analysis are critical [8]. Reflex tissue triaging, where molecular requirements are anticipated during diagnostic evaluation, minimizes the need for repeat biopsies and accelerates testing workflows [11].

### 6.2. Testing Algorithms for Resected NSCLC Specimens

Structured algorithms can guide molecular testing for resected specimens, ensuring that essential biomarkers are evaluated systematically. Current recommendations emphasize initial testing for common alterations [11] such as EGFR, ALK, and KRAS, followed by broader panels that capture less frequent but actionable drivers including RET, ROS1, and NTRK [10]. Reflex testing initiated by pathology departments has been shown to reduce delays, particularly in early-stage disease where adjuvant therapy decisions may be time-sensitive [14]. Integration of testing algorithms into institutional workflows promotes consistency and reduces variability in care.

### 6.3. Integrating NGS into Standard Practice

NGS platforms are increasingly considered the most efficient approach for comprehensive biomarker testing, given their ability to interrogate multiple targets simultaneously. Adoption into standard practice requires investment in infrastructure, training, and bioinformatics support [9,13]. To maximize utility, institutions should establish clear policies regarding when NGS should be prioritized over sequential single-gene assays [6,7]. Incorporating NGS into multidisciplinary tumor boards ensures that complex genomic findings are appropriately interpreted and translated into actionable clinical strategies [5].

### 6.4. Enhancing Access to Targeted Therapies

The value of molecular testing is realized only when patients can access therapies matched to their biomarkers [6]. Practical steps include aligning testing with approved drug indications, securing reimbursement for guideline-recommended assays, and ensuring timely turnaround of results [6]. Expanding access also requires advocacy for policy-level reforms to reduce disparities between high- and low-resource settings [5]. Liquid biopsy can help extend testing to patients without adequate tissue and support rapid clinical decision-making [3]. In resected NSCLC, the clinical value of molecular testing depends not only on analytical performance but also on timely access to biomarker-directed treatment pathways [14].

## 7. Limitations of This Review

This review has several limitations that should be acknowledged. It integrates technical, clinical, and health-system literature characterized by heterogeneity in study design, patient populations, testing platforms, and clinical endpoints, which may limit direct comparability across studies and reduce the generalizability of some conclusions. In addition, the field of molecular testing in NSCLC is evolving rapidly, particularly with respect to next-generation sequencing, liquid biopsy, artificial intelligence-assisted diagnostics, and targeted therapies, and some aspects of the present discussion may therefore require future updating. Several real-world barriers discussed in this review, including reimbursement, accessibility, turnaround time, and interdisciplinary coordination, are strongly influenced by local healthcare systems, institutional infrastructure, and national policy environments, which may limit the universal applicability of these observations. Furthermore, because this review intentionally covers a broad range of topics, some areas are discussed in less depth than they would be in a focused review dedicated to a single domain. In addition, while particular emphasis is placed on TP53, KRAS, EGFR, and ALK because of their biological and clinical relevance, other actionable alterations relevant to contemporary NSCLC management are not discussed in equal depth. Finally, although this review addresses the prognostic and therapeutic relevance of molecular testing and includes selected outcome-based examples from resected disease, it does not provide pooled quantitative estimates of survival benefit or treatment effect specifically for resected NSCLC. These limitations should be considered when interpreting the conclusions of the present work.

## 8. Conclusions

Molecular testing has become an integral component of precision oncology in non-small cell lung cancer, particularly as the number of clinically actionable genomic alterations continues to expand. Despite major advances in molecular diagnostics and targeted therapies, the effective implementation of comprehensive testing in resected NSCLC specimens remains challenged by technical limitations, sample quality issues, logistical delays, and disparities in access across healthcare systems.

This review highlights the critical role of optimized tissue handling, appropriate test selection, and interdisciplinary collaboration in maximizing the clinical utility of molecular profiling. The integration of next-generation sequencing with conventional diagnostic methods, along with emerging approaches such as liquid biopsy, multi-omics profiling, and artificial intelligence, offers promising strategies to overcome current barriers. As molecular testing increasingly informs adjuvant and perioperative treatment decisions, standardized workflows and equitable access to advanced diagnostics will be essential. Addressing these challenges may enable more effective personalization of therapy and support improved outcomes for patients with resected NSCLC. Accordingly, the present review is intended to provide a broad clinically oriented overview rather than an exhaustive analysis of each individual domain within molecular testing in resected NSCLC. As molecular diagnostics and targeted treatment strategies continue to evolve, ongoing refinement of testing workflows and broader access to precision-guided care will remain essential in resected NSCLC.

## Figures and Tables

**Table 1:** Molecular alterations with a frequency of more than 4% in all cohorts.

Gene Alteration	Frequency (%)
TP53	52.9
KRAS	20.0
EGFR	8.6
STK11	8.6
PIK3CA	7.1
CDKN2A	7.1
ALK	5.7
ERBB2 (HER2)	4.3
RB1	4.3

Data reproduced and adapted from [4].

## Data Availability

No new data were created or analyzed in this study. Data sharing is not applicable to this article.

## Abbreviations

NSCLC	non-small cell lung cancer;
NGS	next-generation sequencing;
IHC	immunohistochemistry;
FISH	fluorescence in situ hybridization;
RT-PCR	reverse transcription polymerase chain reaction;
FFPE	formalin-fixed paraffin-embedded;
PD-L1	programmed death-ligand 1;
TKI	tyrosine kinase inhibitor;
EGFR	epidermal growth factor receptor;
ALK	anaplastic lymphoma kinase;
ROS1	ROS proto-oncogene 1;
KRAS	Kirsten rat sarcoma viral oncogene homolog;
BRAF	B-Raf proto-oncogene;
MET	mesenchymal–epithelial transition factor;
RET	rearranged during transfection;
HER2	human epidermal growth factor receptor 2;
NTRK	neurotrophic tyrosine receptor kinase.
